# Reassessing potential economic value and health impact of effective *Shigella* vaccines

**DOI:** 10.2471/BLT.23.290163

**Published:** 2023-12-11

**Authors:** William P Hausdorff, John D Anderson, A Louis Bourgeois, Allison Clifford, Jessica A Fleming, Farzana Muhib, Clint Pecenka, Chloe Puett, Mark S Riddle, Suzanne Scheele, Karoun H Bagamian

**Affiliations:** a Center for Vaccine Innovation and Access, PATH, 455 Massachusetts Avenue NW, Washington, D.C. 20001, United States of America (USA).; b West Virginia University, Morgantown, West Virginia, USA.; c PATH, Seattle, Washington, USA.; d Stony Brook University, Stony Brook, New York, USA.; e University of Nevada, Reno, Nevada, USA.; f Bagamian Scientific Consulting LLC, Gainesville, Florida, USA.

## Abstract

The gram-negative bacterium *Shigella* is a leading cause of diarrheal morbidity and mortality in children in low- and middle-income countries. Several promising vaccine candidates are in late stages of clinical development against this increasingly antibiotic-resistant pathogen. However, considering the increasingly crowded and costly paediatric immunization schedule, and likely advent of other important new vaccines, it is unclear whether introduction of a *Shigella* vaccine would represent a high priority for international agencies or health ministries in low- and middle-income countries. To determine whether there is a compelling public health value proposition for a *Shigella* vaccine, we used the World Health Organization’s Full Value of Vaccine Assessment analytic framework and formulated five broad scientific, policy, economic and commercial-related propositions regarding the development of a *Shigella* vaccine. We also explored the current regulatory, clinical, policy and commercial challenges to a *Shigella*-containing combination vaccine development and adoption. Through a series of literature reviews, expert consultations, social science field studies and model-based analyses, we addressed each of these propositions. As described in a series of separate publications that are synthesized here, we concluded that the economic and public health value of a *Shigella* vaccine may be greater than previously recognized, particularly if it is found to also be effective against less severe forms of diarrheal disease and childhood stunting. The decision by pharmaceutical companies to develop a standalone vaccine or a multipathogen combination will be a key factor in determining its relative prioritization by various stakeholders in low- and middle-income countries.

## Résumé

La bactérie à Gram négatif *Shigella* est l’une des principales causes de morbidité et de mortalité diarrhéiques chez les enfants des pays à revenu faible et intermédiaire. Plusieurs candidats vaccins prometteurs sont en phase avancée de conception clinique contre cet agent pathogène qui connaît une antibiorésistance croissante. Toutefois, compte tenu du calendrier de vaccination pédiatrique de plus en plus chargé et coûteux et de l’arrivée probable d’autres nouveaux vaccins importants, il n’est pas certain que la mise sur le marché d’un vaccin contre *Shigella* constitue une priorité élevée pour les agences internationales ou les ministères de la Santé des pays à revenu faible ou intermédiaire. Pour déterminer l’existence d’un intérêt convaincant en matière de santé publique pour un vaccin contre *Shigella*, nous avons utilisé le cadre analytique du cadre d’évaluation de la valeur totale des vaccins de l’Organisation mondiale de la santé et formulé cinq propositions scientifiques, politiques, économiques et commerciales générales concernant la conception d’un vaccin contre *Shigella*. Nous avons également étudié les défis en matière réglementaire, clinique, politique et commerciale qui se posent actuellement à la mise au point et à l’adoption d’un vaccin combiné contenant des *Shigella*. Nous avons abordé chacune de ces propositions au moyen d’une série d’analyses documentaires, de consultations d’experts, d’études de terrain en sciences sociales et d’analyses basées sur des modèles. Comme décrit dans une série de publications distinctes résumées ici, nous avons conclu que la valeur économique et sur le plan de la santé publique d’un vaccin contre *Shigella* pourrait être plus importante que ce qui était considéré précédemment, en particulier s’il s’avère que ce vaccin s’avère également efficace contre les formes moins sévères de maladies diarrhéiques et de retard de croissance chez l’enfant. La décision d’entreprises pharmaceutiques de mettre au point un vaccin autonome ou une combinaison de plusieurs agents pathogènes sera un facteur clé dans la détermination de sa priorité relative par les différentes parties prenantes dans les pays à revenu faible et intermédiaire.

## Resumen

La bacteria gramnegativa *Shigella* es una de las principales causas de morbilidad y mortalidad por diarrea en niños de países de ingresos bajos y medios. Varias vacunas candidatas y prometedoras se encuentran en las últimas fases de desarrollo clínico contra este patógeno cada vez más resistente a los antibióticos. Sin embargo, teniendo en cuenta el esquema de inmunización pediátrica, cada vez más saturado y costoso, y la probable llegada de otras vacunas nuevas importantes, no está claro si la introducción de una vacuna contra la *Shigella* representaría una alta prioridad para los organismos internacionales o los ministerios de salud de los países de ingresos bajos y medios. Para determinar si existe una propuesta de valor de salud pública convincente para una vacuna contra la *Shigella*, utilizamos el marco de análisis Full Value of Vaccine Assessment de la Organización Mundial de la Salud y formulamos cinco amplias propuestas científicas, políticas, económicas y comerciales relacionadas con el desarrollo de una vacuna contra la *Shigella*. También exploramos los actuales desafíos reglamentarios, clínicos, políticos y comerciales para el desarrollo y la adopción de una vacuna combinada que contenga *Shigella*. Mediante una serie de revisiones bibliográficas, consultas a expertos, estudios de campo de ciencias sociales y análisis basados en modelos, abordamos cada una de estas proposiciones. Como se describe en una serie de publicaciones separadas que se sintetizan aquí, llegamos a la conclusión de que el valor económico y de salud pública de una vacuna contra la *Shigella* puede ser mayor de lo que se reconocía anteriormente, en particular si se descubre que también es eficaz contra formas menos graves de enfermedad diarreica y retraso del crecimiento infantil. La decisión de las empresas farmacéuticas de desarrollar una vacuna independiente o una combinación multipatógena será un factor clave a la hora de determinar su prioridad relativa por parte de las diversas partes interesadas en los países de ingresos bajos y medios.

## ملخص

تعد بكتيريا شيغيلا سلبية الجرام سببًا أساسيًا للأمراض والوفيات نتيجة للإسهال لدى الأطفال في الدول ذات الدخل المنخفض والدخل المتوسط. هناك العديد من اللقاحات المرشحة الواعدة في مراحل متأخرة من التطوير الإكلينيكي ضد هذا العامل المسبب للمرضالمقاوم للمضادات الحيوية بشكل متزايد. ومع ذلك، ومع الوضع في الاعتبار جدول تحصين الأطفال المزدحم والمكلف بشكل متزايد، والظهور المحتمل للقاحات جديدة مهمة أخرى، فمن غير الواضح ما إذا كان طرح لقاح شيغيلا سيمثل أولوية مرتفعة للهيئات الدولية، أو وزارات الصحة في الدول ذات الدخل المنخفض والدخل المتوسط. لتحديد ما إذا كان هناك اقتراح منطقي لقيمة الصحة العامة للقاح شيغيلا، فقد استخدمنا إطار العمل التحليلي لتقييم القيمة الكاملة للقاحات التابع لمنظمة الصحة العالمية، وقمنا بصياغة خمسة اقتراحات واسعة النطاق، علمية وسياسية واقتصادية وتجارية تتعلق بتطوير لقاح شيغيلا . لقد استكشفنا أيضًا التحديات التنظيمية، والإكلينيكية، والسياسية، والتجارية الحالية التي تواجه تطوير واعتماد لقاح مركب يحتوي على شيغيلا. ومن خلال سلسلة من مراجعات الأدبيات، ومشاورات الخبراء، والدراسات الميدانية للعلوم الاجتماعية، والتحليلات القائمة على النماذج، فقد تناولنا كلًا من هذه الاقتراحات. كما هو موضح في سلسلة من المنشورات المنفصلة التي تم تجميعها هنا، فقد استنتجنا أن القيمة الاقتصادية والصحية العامة للقاح شيغيلا قد تكون أكبر مما تم إدراكه سابقًا، وخاصة إذا تم اكتشاف أنه فعال أيضًا ضد الأشكال الأقل خطورة من أمراض الإسهال، والتقزم في مرحلة الطفولة. إن القرار الذي تتخذه شركات الأدوية بتطوير لقاح مستقل، أو مجموعة متعددة من العوامل المسببة للأمراض، سيكون عاملاً رئيسياً في تحديد أولوياتها النسبية بواسطة مختلف أصحاب المصلحة في الدول ذات الدخل المنخفض والدخل المتوسط.

## 摘要

革兰氏阴性菌*志贺氏菌*是中低收入国家导致儿童腹泻发病和死亡的主要原因。针对这种对抗生素耐药性日益增强的病原体，几种有希望的候选疫苗正处于临床开发的后期阶段。然而，考虑到种类越来越多且价格越来越高的儿科免疫接种计划，以及可能出现的其他重要新疫苗，目前尚不清楚引入*志贺氏菌*疫苗是否会成为国际机构或中低收入国家卫生部的高度优先事项。为了确定*志贺氏菌*疫苗是否具有令人信服的公共卫生价值主张，我们使用了世界卫生组织的疫苗全面价值评估分析框架，并就*志贺氏菌*疫苗的开发提出了五项广泛的科学、政策、经济和商业相关命题。我们还探讨了目前含有*志贺氏菌*的联合疫苗在开发和采用方面所面临的监管、临床、政策和商业挑战。通过参阅一系列文献综述、进行专家咨询、开展社会科学领域的研究和基于模型的分析，我们解决了所有命题。正如本文综合的一系列独立出版物中所述的情况，我们得出的结论是，*志贺氏菌*疫苗的经济和公共卫生价值可能比以前认识到的更大，特别是当发现它也能有效干预不太严重的腹泻病和儿童发育迟缓。制药公司开发独立疫苗还是含有多种病原体的联合疫苗，这一决定将是中低收入国家各利益攸关方确定其相对优先级的一个关键因素。

## Резюме

Грамотрицательная бактерия *Shigella* является одной из основных причин возникновения кишечных диарейных инфекций и смертности среди детей в странах с низким и средним уровнем доходов. Несколько перспективных образцов вакцин находятся на поздних стадиях клинической разработки против этого все более устойчивого к антибиотикам патогенного микроорганизма. Однако с учетом все более плотного и дорогостоящего графика иммунизации детей и вероятного появления других важных новых вакцин неясно, будет ли внедрение вакцины против *Shigella* приоритетным для международных агентств или министерств здравоохранения в странах с низким и средним уровнем доходов. Чтобы определить, существует ли убедительное предложение по повышению ценности вакцины против бактерии *Shigella* для общественного здравоохранения, использовалась аналитическая система Всемирной организации здравоохранения «Оценка полной ценности вакцины» и были сформулированы пять широких научных, политических, экономических и коммерческих предложений, касающихся разработки вакцины против бактерии *Shigella*. Также были изучены существующие регуляторные, клинические, политические и коммерческие проблемы, препятствующие разработке и внедрению комбинированной вакцины с содержанием бактерии *Shigella*. Авторы рассмотрели все эти предложения с использованием серии обзоров литературы, консультаций с экспертами, полевых исследований в области социальных наук и анализа на основе моделей. Основываясь на серии отдельных публикаций, обобщенных в настоящем документе, был сделан вывод о том, что экономическая ценность вакцины против бактерии *Shigella* для общественного здравоохранения может быть выше, чем признавалось ранее, особенно если окажется, что она также эффективна против менее тяжелых форм острых кишечных инфекций и задержки роста в детском возрасте. Решение фармацевтических компаний о разработке отдельной вакцины или многопатогенной комбинации станет ключевым фактором, определяющим их относительную приоритетность для различных заинтересованных сторон в странах с низким и средним уровнем доходов.

## Introduction

In humans, the gram-negative bacterium *Shigella* causes moderate to severe diarrheal disease, including dysentery.^1^ Shigellosis is also increasingly associated with other chronic and long-term conditions,^1^^,^^2^ such as growth faltering or stunting in children,^3^^,^^4^ and irritable bowel syndrome^5^ or reactive arthritis in adults.^6^ However, whether these associations represent causal relationships has not yet been rigorously assessed.

After rotaviru*s*, *Shigella* is the leading cause of diarrheal disease-related mortality among children younger than five years in low- and middle-income countries.^7^ Since transmission is associated with unclean water and poor sanitation conditions, high-risk populations other than children include travellers and military personnel staying in unsanitary settings.^1^ The global estimate of annual *Shigella* deaths in children younger than five years decreased from 600 000 in the 1990s^8^ to current estimates of 28 000–64 000.^7^^,^^9^^,^^10^ The observed reduction may be attributable to several factors, including enhancements in living conditions, the implementation of health interventions not related to vaccines, and the advancement of disease monitoring through the use of molecular diagnostic techniques. However, treatment is becoming more complex, costly and time intensive because of *Shigella’s* increasing resistance to antibiotics.^11^ Improved diagnostics have shown that antibiotic-resistant *Shigella* is causing more cases of acute watery diarrhoea (both severe and mild types) than previously recognized. This revelation has led researchers to revise previous estimates of its etiological importance in both moderate to severe diarrhoea cases, and the more common but less severe diarrhoea.^4^^,^^12^

A few promising *Shigella* vaccine candidates are currently in Phase II or starting Phase III clinical trials with projected availability within the next 5 years.^1^^,^^13^ The question, however, is whether these vaccines would be sufficiently prioritized within broad policy recommendations to influence their adoption into immunization programmes in countries where *Shigella* is endemic.^14^ This uncertainty underscores the need for a broader methodological approach to evaluate the potential benefits of a *Shigella* vaccine. To do this, we adopted the World Health Organization’s (WHO) Full Value of Vaccine Assessment framework^15^ to assess the value of a *Shigella* vaccine for a broad range of health, societal and economic benefits at both the individual and population levels. 

## Assessing the value

The vaccine assessment framework^5^^,^^15^ highlights the importance of a general understanding of how different aspects of introducing a new vaccine may influence the view of immunization advisory committees, health ministries and health-care providers. To gain further understanding, we formulated five scientific, policy, economic and commercial-related questions: (i) What is the evidence for the association between shigellosis, diarrhoeal disease and stunting? (ii) To what extent could a vaccine avert morbidity and mortality, health costs and larger economic consequences due to acute and long-term effects of *Shigella*? (iii) What is the perceived value of a *Shigella* vaccine to policy-makers and health-care workers in low- and middle-income countries, as well as travel medicine providers and military health policy-makers? (iv) What are the challenges and opportunities to developing and introducing a *Shigella*-containing combination vaccine? and (v) What could be anticipated demand for a standalone *Shigella* vaccine by low- and middle-income countries as well as travellers and military? 

To answer these questions we searched the literature, consulted experts, conducted social science field research, modelled health impacts and economic outcomes, and constructed demand forecasts. The results, described in a series of separate publications, are synthesized below.

### Association with stunting

Several studies, including two large, multicountry analyses in low- and middle-income countries,^4^^,^^12^^,^^16^^–^^20^ have consistently observed statistically significant associations between moderate to severe diarrhoea, less severe diarrhoea and diminished childhood growth such as stunting. Furthermore, studies have specifically linked *Shigella*-attributable moderate and severe diarrhoea in infants and toddlers with linear growth faltering as early as 2–3 months after the initial episode; in one study, linear growth faltering was documented to persist for at least 3 years post-infection.^16^^,^^21^ Furthermore, children aged 12–24 months with confirmed shigellosis who were subsequently treated with antibiotics exhibited a fourfold decrease in linear growth faltering when compared to their untreated counterparts.^18^

Although the incidence of *Shigella*-attributable diarrhoea in children aged 18–59 months is higher than in infants younger than 12 months,^16^ an etiology-agnostic analysis suggests that the stunting-related consequences of moderate to severe diarrhoea appear greater at younger ages.^22^ Taken together, these data favour delivery of the first dose of a two-dose series by 6 months of age.^17^

A plausible estimate of the effect of a single *Shigella-*attributable diarrhoea episode would be a 0.03 length-for-age z-score decrement for children younger than 2 years, with a cumulative upper bound for all *Shigella* episodes of a 0.15 length-for-age z-score decrement.^17^ In contrast, the association of asymptomatic shigellosis with stunting is less well established. Given the current lack of evidence that *Shigella* vaccines could fully prevent infections (i.e. induce sterilizing immunity), we considered it too early to include asymptomatic shigellosis in vaccine modelling efforts.^17^

Hypotheses regarding the underlying mechanisms by which *Shigella* and other entero-invasive pathogens may lead to stunting involve intestinal tract disruption, including the inflammation and intestinal tract atrophy that are hallmarks of environmental enteric dysfunction.^17^ Uncertainties remain whether a vaccine that is effective against shigellosis but not infection itself would affect these underlying mechanisms. Nonetheless, parenterally administered conjugate vaccines targeting other pathogens such as pneumococcus have demonstrated a substantial impact on bacterial mucosal colonization.^23^ There is some evidence to support that *Shigella* vaccines could act to alter the density or anatomical location of intestinal colonization and potentially reduce chronic inflammation.^24^ Vaccine probe studies are needed to directly determine the magnitude, if any, of the influence of *Shigella* vaccines on linear growth faltering or stunting.

### Reduction in adverse outcomes

Two recent analyses^25^^,^^26^ addressed the extent to which a vaccine could mitigate morbidity and mortality, reduce health-care expenses, and alleviate broader economic repercussions resulting from both the acute and long-term effects of *Shigella*. In the first analysis,^25^^,^ we developed a simulation model to evaluate *Shigella*-attributable less severe diarrhoea-associated episodes and childhood stunting in 102 low- and middle-income countries. The vaccine's hypothetical characteristics and delivery schedule were based on *WHO preferred product characteristics for vaccines against Shigella*.^27^

**Fig. 1:**
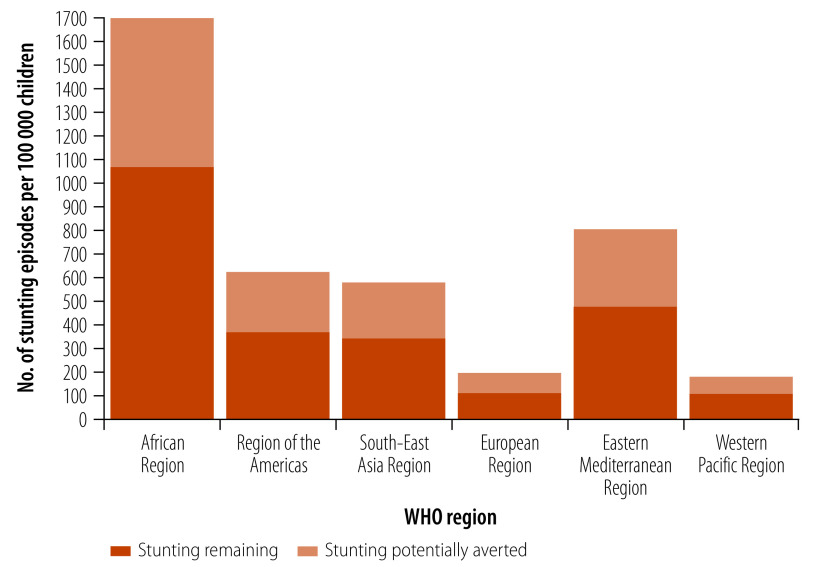
Projected impact on *Shigella*-attributable stunting episodes due to the introduction of an effective *Shigella* vaccine, by WHO region

**Fig. 2:**
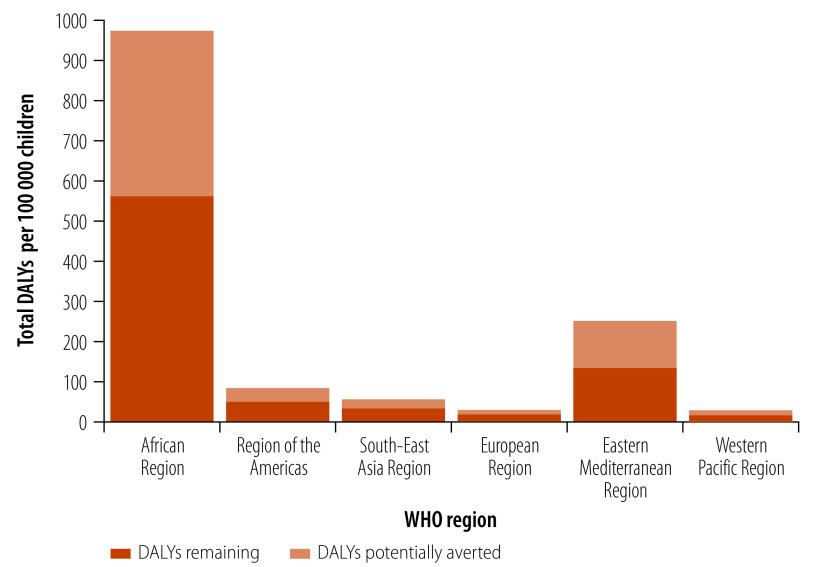
Disability adjusted life years potentially averted due to the introduction of an effective *Shigella* vaccine by WHO region

DALY: disability adjusted life years; WHO: World Health Organization.

According to our simulation (without vaccination) over a 20-year period (2025–2044), *Shigella* is estimated to be responsible for 109 million stunting episodes, 1.4 million deaths and incur health-care expenses exceeding 11 billion United States dollars (US$). This finding suggests that an effective *Shigella* vaccine could potentially avert 43 million stunting cases, nearly 600 000 deaths and US$ 4.4 billion in health-care costs if available over the same 20 year period.^25^ Fig. 1 illustrates the regional variability in the numbers of stunting episodes averted by vaccination and Fig. 2 shows how projected decreases in stunting and other health effects would translate into averted disability adjusted life years (DALYs). 

WHO: World Health Organization.

Data source: Adapted from Anderson et al.^25^

Data source: Adapted from Anderson et al.^25^

The average cost per DALY averted is US$ 849, with the vaccine being more cost-effective in the African Region (US$ 161 per DALY averted), low-income countries (US$ 143 per DALY averted) and countries eligible for support from Gavi, the vaccine alliance (US$ 308 per DALY averted). One main contributor to the lower incremental cost-effectiveness ratios^25^ relative to prior analyses^28^ was the inclusion of less severe diarrhoea and less severe diarrhoea-attributable stunting; a second was the assumption that in Gavi-eligible countries, a two-dose vaccine (at US$ 2 per dose) might become available rather than the previously assumed three-dose vaccine at US$ 3.30 per dose.^28^ We based our price estimates on the current prices of monovalent typhoid conjugate vaccines. 

While the simulation suggests that *Shigella* vaccination may approach the cost-effectiveness of other routinely administered childhood vaccines,^25^ it still probably underestimates the true economic value of the vaccine. This underestimation is because childhood linear growth faltering and stunting can ultimately influence adult height, which has been shown to be associated with differentials in labour-market wages across various low- and high-income settings.^26^^,^^29^ The extent to which this latter association (termed the height premium) is causal remains unclear and complex, but has been previously cited as a means to quantify future economic productivity benefits of various nutritional interventions.^29^

Second, we developed a benefit-cost analysis model^26^ to estimate the potential productivity benefits of a vaccine that could reduce *Shigella*-attributable linear growth faltering relative to the net costs of a vaccine programme. This analysis^26^ used the same cost, vaccine efficacy and epidemiological assumptions as the simulation model,^25^ and relied on estimates of *Shigella* impact on growth faltering. We then applied a literature-based coefficient that translates each country-specific change in childhood length-for-age z-score to a change in adult height. Given variability in reported height premium values, we first conducted a meta-analysis of the economic literature to allow us to populate our model with a reasonable estimate.^29^

Using a discount rate of 3%, we determined that the benefit-cost ratio was 8.5 across all 102 low- and middle-income countries, indicating that the monetary value of benefits is higher than the costs associated with *Shigella* vaccination (Table 1). Benefit-cost ratios were above one (parity) in all WHO regions; and highest in South-East Asia Region (15.3), Region of the Americas (10.9), African Region (8.1) and Gavi-eligible countries (14.5). We found these benefit-cost ratios to be highly robust to various sensitivity analyses. Even at only 10% effectiveness in preventing stunting, a *Shigella* vaccine was still predicted to provide more monetary value than its cost in most WHO regions.^26^

**Table 1:** Benefit-cost ratios including productivity benefits of preventing *Shigella*-attributable growth faltering through vaccination

Country group	Benefit-cost ratios, by discounting rate	
	3%	6%
African Region	8.11	2.53
Region of the Americas	10.85	3.97
Eastern Mediterranean Region	2.69	0.91
European Region	4.65	1.61
South-East Asia Region	15.26	4.27
Western Pacific Region	5.68	1.73
Gavi-eligible countries (*n*=53)	14.45	4.11
All low- and middle-income countries	8.53	2.58

Note: Assumed average retirement age at 64 years and with 3% or 6% annual discounting rates.

Data source: Puett et al.^26^

We note that neither model (simulation or benefit-cost analysis) estimated the impact of an effective *Shigella* vaccine on antimicrobial use and disease attributed to antibiotic-resistant *Shigella* strains; however modelling efforts regarding these aforementioned impacts are currently underway at WHO.^30^^–^^31^

### Perceived value of a vaccine

We conducted a mixed-methods study using semi-structured individual interviews with 89 national policy-makers and health-care providers in Burkina Faso, Ghana, Kenya, Nepal and Viet Nam, as well as with regional immunization and enteric disease experts at the Regional Office for the Americas in 2021–2022, to assess the perspectives of stakeholders in low- and middle-income countries on the introduction of a *Shigella* vaccine.^32^ We chose these five countries to represent known heterogeneity in local shigellosis burden, previous experience with diarrheal disease vaccines, stunting prevalence, and Gavi financing eligibility.^32^ We queried their perceptions of the relative importance of diarrheal disease, stunting, antimicrobial resistance and *Shigella* itself. We then explored a *Shigella* vaccine’s potential attractiveness using the vaccine attributes and delivery schedule from *WHO preferred product characteristics for vaccines against Shigella*.^27^

The low- and middle-income countries mixed-methods study^32^ revealed that diarrhoeal disease awareness was high among study participants. However, most respondents did not rank *Shigella* as a serious health concern for children younger than 5 years and, in the absence of additional information, more than half considered a *Shigella* vaccine to be a medium or low priority, with national stakeholders generally assigning a lower priority than health-care providers. Some of the reasons participants cited were lower *Shigella*-attributable mortality burden compared to other vaccine-preventable diseases, and large number of immunizations children currently receive. 

**Fig. 3:**
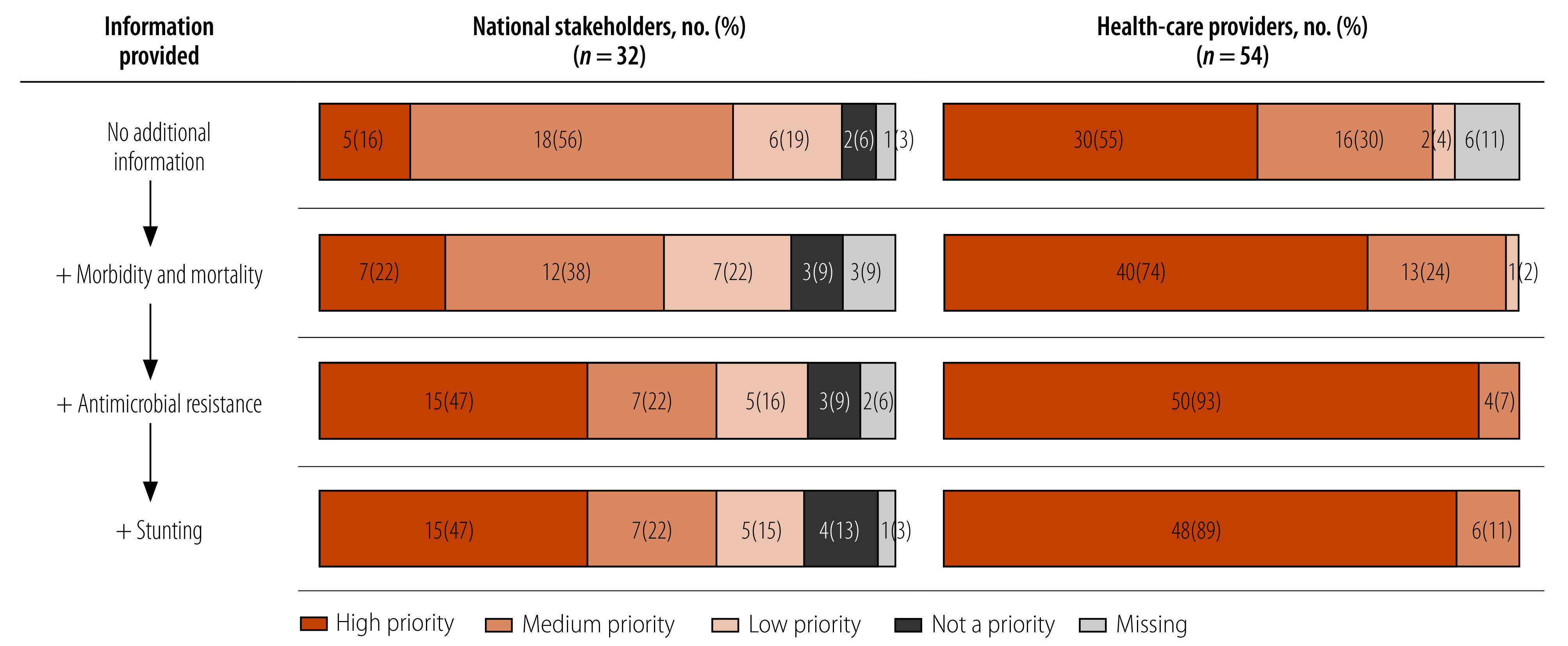
Stakeholders and health-care providers perceived priority of introducing a *Shigella* vaccine into the national immunization programme, low- and middle-income countries 2021–2022

Note: Each participant was first asked to characterize the priority of *Shigella* vaccine introduction, and then asked again three times following receipt of progressively more information. Participants were from Burkina Faso, Ghana, Kenya, Nepal, Viet Nam and the World Health Organization’s Regional Office for the Americas.

However, when asked to consider a *Shigella* vaccine’s potential ability to reduce antimicrobial resistance or stunting burden, more than two thirds of each group deemed the vaccine's introduction to be of medium or high priority (Fig. 3). Their strong concerns about stunting per se were especially evident in their responses to our open-ended survey questions.^32^ Here stakeholders called for more research into country-specific burden and robust evidence of the long-term health impacts of a *Shigella* vaccine.^32^

Data source: Adapted with permission from Fleming et al.^32^

**Fig. 4:**
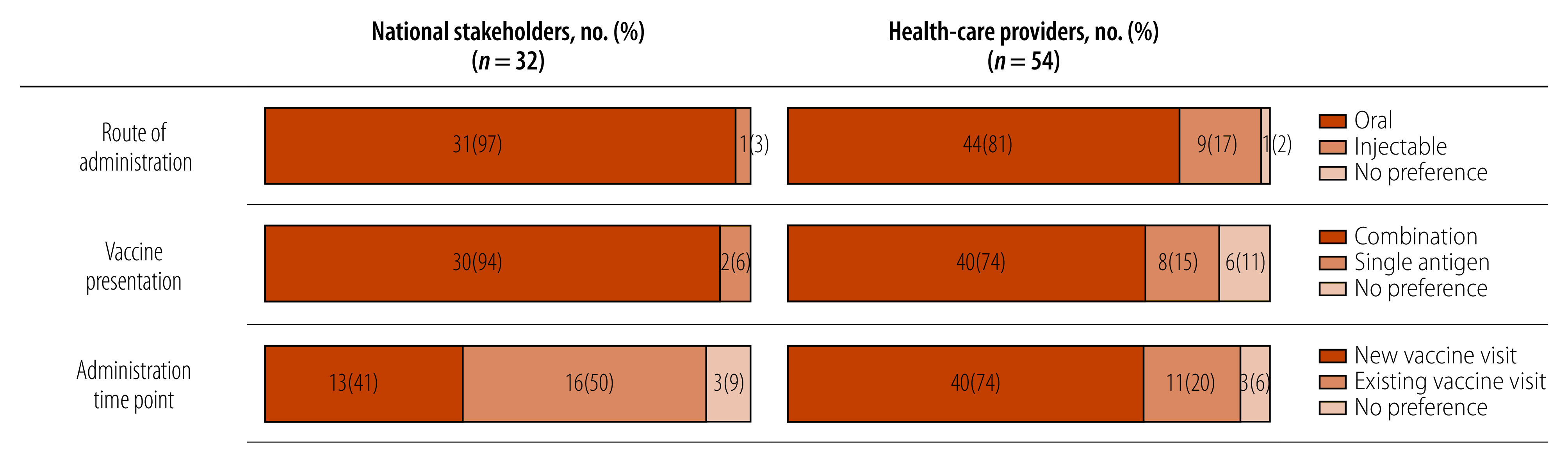
Stakeholders and health-care providers preferred *Shigella* vaccine attributes, low- and middle-income countries, 2021–2022

Note: Participants were from Burkina Faso, Ghana, Kenya, Nepal, Viet Nam and the World Health Organization’s Regional Office for the Americas.

In terms of vaccine delivery, study participants from low- and middle-income countries strongly preferred oral or combination *Shigella* vaccines over injectable or single-antigen presentations, citing greater perceived community acceptability and uptake (Fig. 4). Health-care providers generally preferred creating a new vaccination visit to administer a *Shigella* vaccine rather than using an existing visit, while national stakeholders' preferences were evenly divided.^32^

Data source: Adapted with permission from Fleming et al.^32^

To better understand the travel medicine recommendation pathway and provider perspectives on *Shigella* vaccines, we interviewed 10 individuals involved in government, WHO vaccination policies and the International Society of Travel Medicine, a recognized clinical practice group of the American Society of Tropical Medicine and Hygiene. We then designed and administered an anonymous web-based survey to 31 travel medicine clinicians.^33^ Military perspectives were gathered from interviews, and written questionnaires administered to 11 experts in vaccine development, deployment-associated vaccine use and military public health practice from Belgium, France, Germany, United Kingdom of Great Britain and Northern Ireland and the United States of America.

Because the vaccine attributes described in *WHO preferred product characteristics for vaccines against Shigella* specifically refer to paediatric populations, we first confirmed that vaccine efficacy levels of 65%–80% against *Shigella* moderate and severe diarrhoea (if demonstrated in human challenge studies)^33^ would be acceptable to travel medicine experts; for the military health policy experts, the required threshold was higher at 80%. As expected, the type of recommendations given by travel medicine providers depends largely on the degree of *Shigella* exposure risk in the proposed travel destination. All agreed that vaccine attractiveness would increase if the vaccine were shown to prevent long-term sequelae and/or was combined with a vaccine targeting typhoid or hepatitis A.^33^

### *Shigella* combination vaccine

In 2022, 34 experts from the academic, industry, philanthropic and global health sectors with varying vaccine-related expertise met to discuss hypothetical parenteral combinations of *Shigella* antigens with three existing vaccines administered to infants.^34^ Experts suggested that a *Shigella* combination vaccine conferring protection against multiple pathogens could offer myriad benefits. Some of the noted benefits include greater acceptance and better timeliness of vaccination than a standalone formulation.^35^^,^^36^ Other benefits could include decreases in cold chain transportation and storage space; fewer syringes; fewer needlestick accidents and other vaccine administration errors. While the cost, timing and risks associated with the development of a combination vaccine would likely be greater than for the standalone components, its potential commercial attractiveness would also be higher.

The experts concluded that the target populations of each component of a *Shigella*-containing combination vaccine should substantially overlap in age-incidence and geographical range. Ideally, protection against each pathogen should require the same number of vaccine doses to avoid superfluous doses. Also desirable is overlap in clinical presentation of the targeted pathogens, which brings additive or synergistic effects on disease and reductions in antimicrobial use. The experts also highlighted the need to avoid enhanced reactogenicity, and to ensure compatibility of any adjuvants or excipients included in a *Shigella*-containing combination vaccine.^34^

One of the fundamental challenges is demonstrating the absence of clinically meaningful immunological interference between the various components of such a combination vaccine in field studies, especially when definitive immune correlates of protection are lacking for multiple antigens. The successful testing and licensure of polyvalent pneumococcal and meningococcal conjugate vaccines were also cited by the experts as an example of two potential models for registration of a *Shigella*-containing combination vaccine. Like a *Shigella*-containing combination vaccine, demonstration of efficacy in a clinical study of each vaccine component (in this case, individual serotypes or serogroups) would be precluded by the reality that, taken separately, each was responsible for very few cases of disease. Yet precise immune correlates of protection were also lacking. While vaccine-induced antibodies that target the capsular polysaccharides of pneumococci and meningococci are believed to provide protection against disease, the specific thresholds required likely vary by serotype and serogroup, and in almost all cases remain undetermined. 

To address these challenges, regulatory agencies and international agencies used innovative licensure criteria to facilitate registration of these vaccines.^34^^,^^37^ These criteria include a reliance on approximate antibody thresholds as immune correlates of protection; and acceptance of the possibility that, for pneumococcal vaccines in particular, the multiple immunological comparisons required could nonetheless result in one or more components missing statistical non-inferiority, yet still allow licensure of the product as a whole. Similar approaches will likely be necessary to allow licensure, in the absence of specific immune correlates, not only of the multiserotype *Shigella* vaccines themselves, but also of multipathogenic *Shigella-*containing vaccine combinations.^34^

In conclusion, experts suggest that limited guidance and/or incentives available to developers of future combination vaccines will hinder their progress, and encouraged re-examination of current policy and recommendation processes to facilitate their development and availability.^34^

### Demand for a standalone vaccine

We created a simple market forecast model to estimate potential demand for a two-dose infant *Shigella* vaccine in 102 low- and middle-income countries from 2027 to 2044. For each country, we used the intervals between vaccine availability and country introduction for three recently introduced paediatric vaccines to categorize the country as an early (introduction less than five years from availability), medium (5 to 10 years) or late adopter (more than 10 years). We used each country's coverage of three doses of diphtheria-tetanus-pertussis as a proxy for *Shigella* dose 1 coverage, and the first dose of measles coverage for dose 2 of the *Shigella* vaccine.^38^ The model assumed 10% wastage and a 25% buffer in the introduction year only. The model also assumed linear adoption of the vaccine over 1 to 4 years based on the size of birth cohort; with a larger birth cohort needing longer uptake time. We estimated traveller and military demand assuming a single dose scenario in the base case, and relied on summary information from publicly available sources on comparator vaccine market size, combined with prospectively-collected survey data from key stakeholders and travel medicine providers.^33^ We assumed pricing to be comparable to that of other traveller vaccines.

**Fig. 5:**
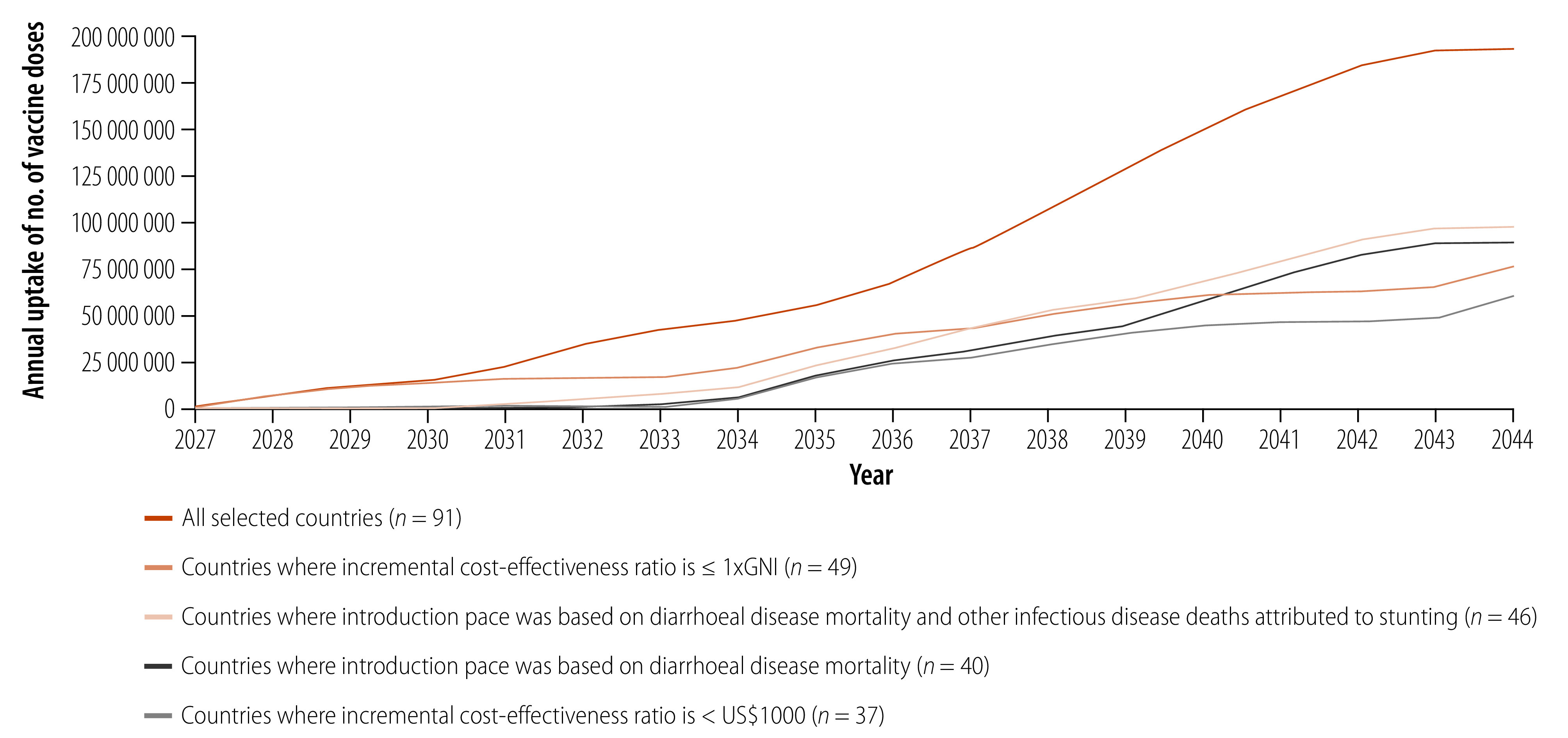
Projected demand forecast for *Shigella* vaccines in 102 low- and middle-income countries, 2027–2044

GNI: gross national income; US$: United States dollars.

Our market forecast model shows that, using the most optimistic scenario where *Shigella* vaccine introduction follows the average historical pace of the broadly recommended rotavirus, pneumococcal conjugate and human papilloma virus vaccines, demand for *Shigella* vaccine in 102 low- and middle-income countries could reach nearly 200 million annual doses by 2044 (Fig. 5). We developed additional scenarios in which countries with moderate (5–20 child deaths per 100 000 persons) and low (less than five deaths per 100 000 persons) annual diarrhoea-associated mortality rates^7^ experienced 1 to 5 or 15 additional years of delay before vaccine introduction, compared to high burden countries (greater than 20 deaths per 100 000 persons). In an alternative scenario, countries would not introduce the vaccine if the incremental cost-effectiveness ratios, expressed as cost over DALYs averted, is either (i) above US$ 1000; or (ii) above the gross national income per capita.^25^ Under these various scenarios, demand estimates ranged from 60 million to 100 million annual doses by 2044.

Finally, we generated demand estimates for single-dose *Shigella* vaccines in the traveller and military markets in high-income countries in Canada and the United States as well as for the European and Western Pacific Regions.^33^ Assuming pre-coronavirus disease 2019 (COVID-19) pandemic travel patterns and volumes, combined annual demand among travellers and military personnel could be over 6 million doses and generate an annual revenue of US$ 576 million (range: US$ 270 million to US$ 898 million). For comparison, the global typhoid vaccine market was estimated at US$ 351 million in 2022 and projected to surpass US$ 884 million by 2030.^33^

## Value proposition

*Shigella* is responsible for a considerable proportion of less severe diarrhoea. Both less and moderate-to-severe diarrhoea have been postulated to contribute to linear growth faltering in young children living in low- and middle-income countries, with potentially profound lifetime effects on wage-earning productivity. Our analyses indicate that the potential health impact and economic value of effective *Shigella* vaccines are markedly enhanced compared to estimates made 5 years ago,^28^ as more current models also take into account the value of preventing less-severe diarrhoea and the long-term economic effects of *Shigella*-associated linear growth faltering. Accordingly, including some measure of growth faltering as a robustly powered study endpoint within future efficacy studies will be critical to understand the true value of a *Shigella* vaccine. Similarly, as stakeholders from low- and middle-income countries highlighted the importance of *Shigella* antimicrobial resistance, we suggest that such studies should also assess the impact of *Shigella* vaccines on resistant *Shigella* disease and antibiotic use.

Our findings highlight the need for a comprehensive approach for *Shigella* vaccine development. We recommend that *Shigella* vaccine developers, funding agencies and policy-makers at both global and national level consider a broad range of potential outcomes. This approach aligns with the Full Value of Vaccine Assessment framework.^15^ Such a holistic review is needed, rather than basing decisions solely on our limited understanding of the immediate consequences of shigellosis. Our findings could also inform the elaboration of future guidance on *Shigella* vaccine preferred characteristics^27^ and target product profiles by WHO^39^ or others.^1^ For those target product profiles, we suggest that the preferred indications for use include prevention of growth faltering in addition to the reduction of *Shigella*-associated antibiotic use, and antibiotic-resistant *Shigella-*attributed or associated disease. The preferred formulation could be a multipathogen combination vaccine; and the preferred schedule could specify that the first dose be given at 6 months of age.

Human vaccine challenge models may prove sufficient to allow licensure of the first *Shigella* vaccines for traveller and military populations in the coming years.^1^ We believe that the substantial demand from travellers and military personnel for a standalone vaccine could further motivate vaccine manufacturers to invest in *Shigella* vaccines for populations in low- and middle-income countries who are most affected by shigellosis. To successfully introduce safe and effective vaccines, we need targeted communication and advocacy at both global and local levels to increase awareness of *Shigella* and its related health impacts.
